# High shear rate propulsion of acoustic microrobots in complex biological fluids

**DOI:** 10.1126/sciadv.abm5126

**Published:** 2022-03-11

**Authors:** Amirreza Aghakhani, Abdon Pena-Francesch, Ugur Bozuyuk, Hakan Cetin, Paul Wrede, Metin Sitti

**Affiliations:** 1 Physical Intelligence Department, Max Planck Institute for Intelligent Systems, 70569 Stuttgart, Germany.; 2 Department of Materials Science and Engineering, Macromolecular Science and Engineering, Robotics Institute, University of Michigan, Ann Arbor, MI 48109, USA.; 3 Institute for Biomedical Engineering, ETH Zürich,, 8092 Zürich, Switzerland.; 4 Electrical and Electronics Engineering Department, Özyegin University, 34794 Istanbul, Turkey.; 5 School of Medicine and College of Engineering, Koç University, 34450 Istanbul, Turkey.

## Abstract

Untethered microrobots offer a great promise for localized targeted therapy in hard-to-access spaces in our body. Despite recent advancements, most microrobot propulsion capabilities have been limited to homogenous Newtonian fluids. However, the biological fluids present in our body are heterogeneous and have shear rate–dependent rheological properties, which limit the propulsion of microrobots using conventional designs and actuation methods. We propose an acoustically powered microrobotic system, consisting of a three-dimensionally printed 30-micrometer-diameter hollow body with an oscillatory microbubble, to generate high shear rate fluidic flow for propulsion in complex biofluids. The acoustically induced microstreaming flow leads to distinct surface-slipping and puller-type propulsion modes in Newtonian and non-Newtonian fluids, respectively. We demonstrate efficient propulsion of the microrobots in diverse biological fluids, including in vitro navigation through mucus layers on biologically relevant three-dimensional surfaces. The microrobot design and high shear rate propulsion mechanism discussed herein could open new possibilities to deploy microrobots in complex biofluids toward minimally invasive targeted therapy.

## Teaser

A high shear rate, acoustically powered microrobotic system is presented for efficient propulsion in complex biological fluids.

## INTRODUCTION

Although the human body contains approximately 60% water (*1*), the biological fluids that compose it are extremely complex. These biofluids are rich in proteins, sugars, mineral ions, and many active biomolecules that alter their mechanical and chemical properties, comprising a broad library of biofluids with a wide range of composition, pH, and viscosities (*2*). The presence of biomolecules in these fluids gives rise to transient molecular interactions that cause shear-dependent behaviors (i.e., viscosity is dependent of shear rate) that deviate from the Newtonian behavior of water (i.e., constant viscosity) (*3*) that limit the mobility of micro- and nanoobjects. Furthermore, ubiquitous biofluids such as blood (which contains red blood cells) or mucus (composed of an interconnected protein network) exhibit additional granular or viscoelastic behaviors, which add enormous complexity to their structure and mechanics. These fundamental differences in the fluid mechanics of complex biofluids present a major scientific challenge in the development of medical microrobots that can operate inside these biofluids toward minimally invasive targeted therapy and other medical applications in deep, hard-to-reach, risky, and unprecedented tight regions in our body.

In recent years, diverse medical microrobots have been designed to navigate inside fluids at the low Reynolds number regime, where the dominance of viscous forces over inertial forces in Newtonian fluids requires symmetry breaking to generate propulsion (*4*–*6*). Inspired by biological microswimmers that use flagella or cilia to create nonreciprocal strokes, a myriad of symmetry breaking microrobot designs [e.g., flexible filament-based (*7*, *8*), helical (*9*), and Janus particle–based (*10*, *11*)] and actuation methods [e.g., magnetic (*11*, *12*), chemical (*13*–*15*), acoustic (*16*, *17*), electrical (*18*, *19*), and light-driven (*20*, *21*)] have been proposed. Although these designs and locomotion mechanisms have been successful in Newtonian fluids, their effective locomotion in biological fluids, such as blood, eye vitreous, and mucus that are complex heterogeneous media with non-Newtonian behaviors (*22*), still remains a challenge. Locomotion in viscoelastic biofluids, such as mucus, presents a particularly more challenging problem since small particles or molecules cannot penetrate the protective viscoelastic mucus layer and cannot reach the underlying epithelial surface (*23*, *24*), thus rendering the microrobot ineffective. Although a few microrobot designs have achieved locomotion in specific biofluids using a chemical surface modification (*25*), time-asymmetric reciprocal strokes (*26*), spontaneous symmetry breaking of magnetic microspheres (*27*), and nanoscale helical propeller designs (*28*), a robust single micrometer-scale robot design capable of fast locomotion in a broad range of complex biofluids is still missing.

Here, we present an acoustically powered microrobot design with a high shear rate propulsion mechanism for locomotion in complex biofluids. Previously, we showed the fast locomotion of bubble-based microrobots, with a regular spherical cavity that enabled bubble entrapment and acoustic propulsion, in Newtonian fluids (*16*). The cavity design proposed in this study includes a double reentrant microstructure, which increases the liquid repellency, enhances the bubble stability, and increases the operational lifetime of the microrobot without any chemical surface modification. We demonstrate the use of high shear rates for effective propulsion in complex biofluids, such as blood and mucus, which was not previously realized. The proposed acoustic microrobot can locally create high shear rate microstreaming flow, which anisotropically deforms the surrounding viscoelastic fluid and enables propulsion. The high shear rate anisotropic microstreaming flows and the nonlinear viscoelastic effects in the fluid switch the locomotion dynamics of acoustic microrobots from surface-slipping mode (occurring in Newtonian fluids) to puller-type propulsion (occurring in viscoelastic, non-Newtonian fluids). Last, we demonstrate the locomotion feasibility of acoustic microrobots on mucus-secreting epithelium cells under in vitro conditions. These acoustic microrobots capable of generating high shear rate propulsion could enable effective cargo (e.g., drug, gene, and imaging contrast agent) delivery (*29*, *30*) in heterogeneous and viscoelastic biofluids in the future.

## RESULTS

### Acoustic microrobot design and fabrication

**Fig. 1.**
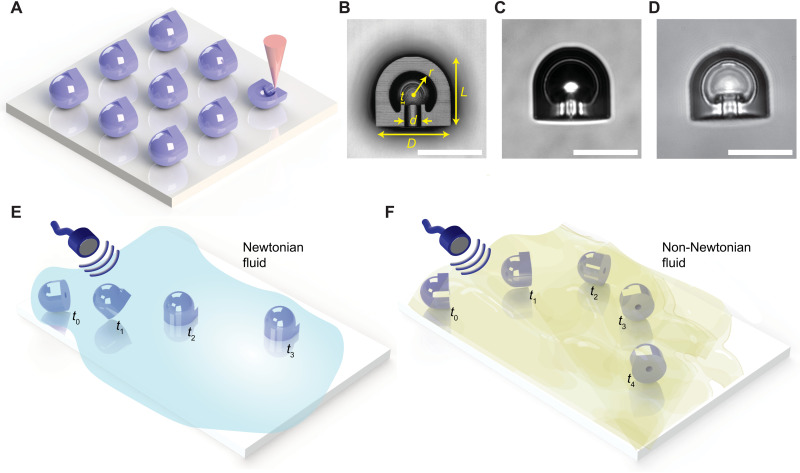
Fabrication, design, and locomotion of the proposed acoustic microrobots. (**A**) Schematics of two-photon polymerization-based 3D microprinting of an acoustic microrobot array on an indium tin oxide (ITO)–coated glass substrate. (**B**) The 3D profile confocal image of a microrobot’s cross section with *D* = 30 μm, *d* = 6 μm, *L* = 27 μm, *r* = 9 μm, and *t* = 2 μm. (**C**) A bright-field microscope image of microrobots with a trapped microbubble in a PBS medium; the trapped gas microbubble is recognized by the darker light intensity. (**D**) A bright-field microscope image of the filled cavity by low surface tension liquid isopropanol. (**E**) Surface-slipping acoustic propulsion of the microrobot in Newtonian fluids, where the orifice of microrobot is aligned perpendicular to the substrate under acoustic actuation. (**F**) Puller-type acoustic propulsion of the microrobot in non-Newtonian fluids, where the orifice is parallel to the substrate under acoustic actuation. The locomotion schematics in (E) and (F) are meant for random starting orientation of microrobots after injection to the medium. Scale bars, 25 μm (B to D).

The acoustic microrobot design consists of a hemispherical shell with a spherical cavity inside. Various microrobot prototypes were three-dimensionally (3D) printed by two-photon polymerization to incorporate microfeatures to trap a microbubble once immersed in a fluid (Fig. 1A). The coating-free cavity design for holding the gas microbubble was inspired by the double reentrant fibers, where the superrepellency of the double reentrant surfaces was previously reported (*31*–*33*). The mushroom-like fibril tips enable liquid repellency without requiring any surface treatment. Since, in the bubble-based acoustic microrobots, the stability of the microbubble is paramount, we integrated these double reentrant features in the spherical cavity to increase the liquid repellency and hence the bubble stability (Fig. 1B). The 2-μm-thick double reentrant edge inside the cavity enabled a robust liquid repellency at the liquid-gas interface. Once the microrobots were immersed in a fluidic medium, an air bubble in the 3D cavity was trapped, as shown in Fig. 1C. However, certain low surface tension fluids, such as isopropanol, could fully wet the cavity surface as shown in Fig. 1D, a standard wetting condition discussed elsewhere (*31*–*33*). The proposed double reentrant cavity design markedly improved the bubble stability for about 48 hours inside phosphate-buffered saline (PBS) solution as compared to 5 to 6 hours in our previous standard spherical cavity design (fig. S1) (*16*). We also tested the microbubble stability in a complex biofluid, such as mucus. The microbubbles inside double reentrant designs were stable again for about 48 hours as compared to 7 to 8 hours in the standard cavity designs (fig. S2). This clearly shows the robustness of the proposed cavity design that enables a longer operation time in heterogenous biofluids. For bubble stability tests inside viscous Newtonian fluids, such as the mixture of glycerol and deionized water (denoted as Gl-DI), we observed a similar stability performance of more than 48 hours for both standard and double reentrant spherical cavity designs (fig. S3). This could be attributed to the higher viscosity of the medium and lower diffusion rate of the gas into the homogenous fluids.

When the acoustic microrobots were injected in a fluidic medium, depending on the rheological property of the fluid, they could have different propulsion modes. In Newtonian fluids, the microrobots showed a surface-slipping motion under ultrasound actuation (Fig. 1E), which we presented recently (*16*). However, in non-Newtonian fluids, we observed puller-type locomotion (Fig. 1F), the reasons of which are explained in the following sections.

### Locomotion in Newtonian and non-Newtonian fluids

**Fig. 2.**
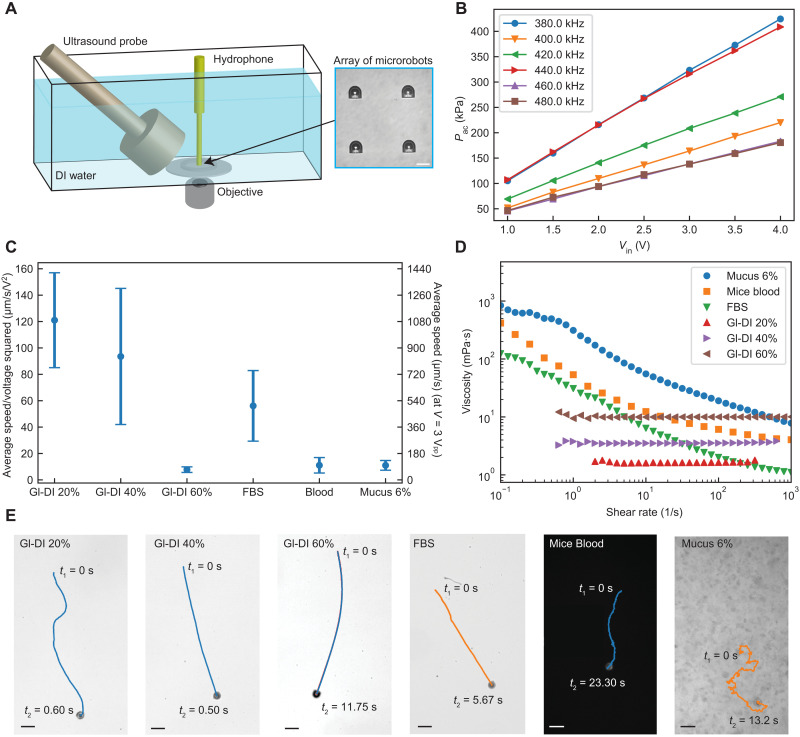
Characterization of the acoustic microrobot locomotion in Newtonian and non-Newtonian fluids. (**A**) The experimental tank setup, where the acoustic pressure was measured using a needle hydrophone close to the microrobots array (inset). Scale bar, 30 μm. (**B**) The experimentally measured acoustic pressures for different input voltages and excitation frequencies. (**C**) The average speed of the microrobots in six different fluids is normalized by the input voltage squared. The average values and the error bars were calculated from five independent locomotion tests for each fluid. The second *y* axis in the diagram gives the average speed value at an input voltage of 3 V_pp_. (**D**) The measured viscosities of six different fluids as a function of the shear rate. (**E**) The trajectory samples of the microrobots in three Newtonian and three non-Newtonian fluids (movie S1). Scale bars, 50 μm.

We evaluated the propulsion of the acoustic microrobot prototypes in different Newtonian and non-Newtonian fluids under ultrasound actuation. The schematics of the experimental setup for characterization of the microrobot propulsion speeds are illustrated in Fig. 2A. Unlike previous studies that use a piezoelectric disk attached to a glass substrate (*8*, *16*, *34*, *35*), we implemented a wireless actuation configuration (shown in Fig. 2A) for a realistic scenario in which an ultrasound probe would be attached to a biological tissue via a coupling gel. For locomotion tests, the acoustic microrobots were injected into a polydimethylsiloxane (PDMS) elastomer cell and actuated by the ultrasound probe in the vicinity of the microbubble resonance frequency, which was around 380 kHz (figs. S4 and S5). The acoustic actuation performance of the double reentrant cavity design was in the same range as the previous spherical cavity design (fig. S5). The range of acoustic pressure amplitudes generated by a planar ultrasound probe in a DI water was measured around 50 to 400 kPa (Fig. 2B) for 1 to 4 V_pp_ (peak-to-peak voltage) input voltage range before amplification. We should note that the acoustic pressure amplitude would be attenuated through soft tissues approximately by a factor of 0.5 to 1.0 dB/cm^−1^ per megahertz (*36*, *37*). For the actuation frequency range of 380 to 480 kHz used in this study, the sound pressure level through 1-cm soft tissue would be attenuated by a factor of 2 to 6%.

We selected three different Newtonian fluid models with different viscosities by preparing Gl-DI mixture with different volume ratios. For non-Newtonian fluids, we selected three biological fluids including fetal bovine serum (FBS), mouse blood, and mucus, all of which have different shear-thinning behaviors (Fig. 2D). The average propulsion speed of microrobots under different driving voltage amplitudes was obtained and normalized by the input voltage square [due to quadratic nonlinearity of acoustic microstreaming (*38*)], as shown in Fig. 2C. The representative microrobot trajectories in different fluids are shown in Fig. 2E and movie S1. For Newtonian fluids, the average instantaneous speed of microrobots reached up to around 1400 μm/s under 3 V_pp_ input driving voltage for 20 and 40% Gl-DI mixture and dropped markedly to around 130 μm/s under 3.5 V_pp_ input voltage for 60% Gl-DI mixture as a result of the increased viscosity. The large SD of normalized speed at lower viscosities could be attributed to the nonlinear response of the microrobots to the acoustic actuation nearby the boundaries (*16*). The physical forces governing the locomotion of the acoustic microrobots are the secondary-order Bjerknes force and the thrust force. The secondary-order Bjerknes force for a free bubble scales as $F_{B}\sim \rho_{f}\langle {\overset{\cdot }{V}}^{2}\rangle /4\pi z^{2}$ with $\overset{\cdot }{V}$ being the rate of volume change and *z* being twice the distance of bubble center from the substrate (*39*, *40*). The thrust force is the result of acoustic microstreaming in the fluid medium and scales as *F*_T_ ~ ϵ^2^ρ_f_*r*^4^*f*^2^, where ϵ is the oscillation amplitude, *r* is the bubble radius, and *f* is the excitation frequency (*38*). The ratio of the two forces, *F*_B_/*F*_T_, determines the propulsion mode of the microrobot. For Gl-DI mixture of 20 and 40%, we observed the surface-slipping motion during propulsion of microrobots, indicating the dominancy of the secondary-order Bjerknes force, which aligns the orifice perpendicular to the substrate. The propulsion speed of microrobots inside 60% Gl-DI mixture with high viscosity of about 10 mPa·s was markedly reduced, and the locomotion orientation of the microrobot was random. This could be attributed to a highly damped oscillation amplitude of the microbubble and a weaker Bjerknes force.

For a shear-thinning FBS, the propulsion speed was considerably high, averaging around 240 μm/s under 2 V_pp_ input voltage. At higher shear rates above 10 s^−1^, the viscosity of FBS dropped to lower than that of 40% Gl-DI mixture, where the propulsion mechanism was observed as surface slipping. For blood and mucus media, the propulsion speed was, on average, around 150 μm/s under 3 V_pp_ input voltage. In terms of propulsion kinematics, the characteristic shear rate $\left(\overset{\cdot }{\gamma }\right)$ has a lower bound of *U*/*D *~ 5 s^−1^ (where *U* is the average speed and *D* is the diameter of the microrobot), which describes the forward locomotion of the microrobot in mucus or blood (*41*). In both blood and mucus cases, the microrobots showed a puller-type propulsion mechanism, where the motion direction was toward the direction of the decreasing viscosity in front of the microrobot. In the presence of non-Newtonian fluids, the Bjerknes force becomes weaker than the thrust force. The interplay between the microstreaming thrust force and weaker Bjerknes force leads to the tilting of the microrobot’s body, causing it to move like a puller microswimmer. Another important effect is the viscoelastic properties, viscosity, and drag of the medium, which changes the oscillation pattern of the microbubble and thus contributes to the propulsion mode switching of the microrobot. After all, it is interesting that the microrobot finds the hydrodynamically favorable path to move inside a heterogenous biofluid, leading to a fast propulsion speed.

We should note that the microrobots could not be operated in shear-thickening fluids, as the high shear rate fluidic flow creates higher viscosity in front of the microrobots, impeding their locomotion. We tested the microrobots in a model shear-thickening fluid composed of 10% (w/w) fumed silica particles suspended in polypropylene glycol (*42*). The viscosity of this fluid oscillated between 15 and 25 Pa·s at the low shear rate and increased with shear rate up to 80 Pa·s (fig. S6). When we actuated the microrobots in this fluid, we did not observe any locomotion as the viscosity is very high at high shear rates. However, while shear-thinning biofluids are abundant in our body (such as blood, serum, and other protein solutions in the human body), shear-thickening biofluids in our body are rare (mostly limited to the polymer and food industries). Therefore, we do not expect that this will limit the operation of the microrobots in a wide range of biofluids in medical applications. Next, to understand the local viscosity gradient nearby the microrobot, we calculate (through numerical simulations) the local shear rate in front of the oscillating microbubble that would indicate the upper bound of $\overset{\cdot }{\gamma }$.

### High shear rate microstreaming flow in Newtonian, shear-thinning, and viscoelastic fluids

**Fig. 3.**
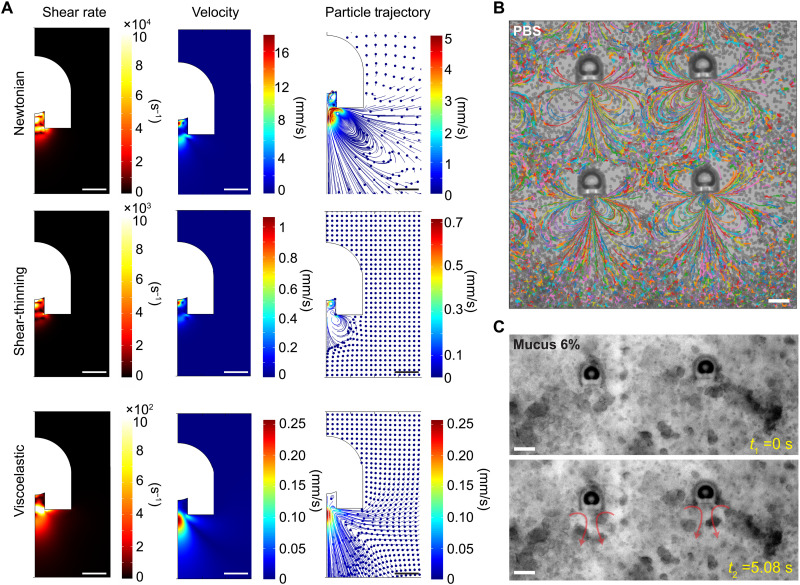
Acoustic microstreaming results of the microrobot in Newtonian and non-Newtonian fluids. (**A**) 2D axisymmetric numerical simulations of the acoustic microstreaming fields, including the flow shear rate, velocity, and particle trajectory for Newtonian (DI water), shear-thinning, and viscoelastic fluid (mucus). The oscillation amplitude and resonance frequency of the microbubble membrane were set to 500 nm and 380 kHz, respectively. (**B**) The experimental trajectories of 2-μm tracer particles in a PBS medium visualizing the microstreaming flow in front of the microrobots, fixed on the substrate, under ultrasound actuation of 380 kHz and 1 V_pp_ (see movie S2). The streamline pattern observed in the experiments matches well with the particle trajectory simulations for the Newtonian fluid in (A). (**C**) Time-lapse images of the acoustic microstreaming in mucus 6%; the mucus microstructure was stretched and displaced in front of the microrobot orifice during the oscillation of the robot microbubble (movie S2). The microstreaming pattern in mucus is similar to the combination of shear-thinning and viscoelastic particle trajectory simulation in (A). Scale bars, 10 μm (A) and 25 μm (B and C).

To understand the propulsion mechanism of the acoustic microrobots in Newtonian and non-Newtonian fluids, we need to predict the acoustic microstreaming field caused by the microbubble oscillation. We have performed 2D axisymmetric simulations to identify the characteristic microstreaming flow generated by the oscillation of the microbubble inside the microrobot’s body. Figure 3A shows the microstreaming shear rate, velocity, and particle trajectory upon acoustic actuation of the microbubble for Newtonian, shear-thinning, and viscoelastic fluids. For modeling the fixated microrobot under acoustic actuation, we defined the microbubble interface as an oscillatory layer with a harmonic velocity profile (*A*ω*e*^*j*ω*t*^), where *A* is the amplitude and ω is the oscillation frequency. The acoustic fields were calculated by solving the first- and second-order perturbation equations (details given in Materials and Methods). The time-averaged streaming velocity is dependent on the fluid rheological behavior. For shear-thinning fluids, we used the Carreau model (*43*) to fit the viscous behavior of the mucus from the rheology experiments as

$$\eta =\eta_{\infty }+(\eta_{0}-\eta_{\infty }){\left[1+{\left(\lambda \overset{\cdot }{\gamma }\right)}^{2}\right]}^{\frac{\left(n-1\right)}{2}}$$ (1)

where the viscosity (η) depends on the shear rate ($\overset{\cdot }{\gamma }$). η_∞_, η_0_, λ, and *n* denote the infinite shear rate viscosity, the zero shear rate viscosity, the relaxation time, and the power index, respectively. For the viscoelastic fluid, we used the Oldroyd-B model to describe the viscoelastic behavior of the fluid under general flow conditions (*44*). The Oldroyd-B model introduces an extra stress tensor (**T**_E_) to the total stress tensor in the Navier-Stokes equation (see Materials and Methods). The extra stress contribution is given by the following constitutive equation (*44*, *45*)

$$T_{E}=2\eta_{S}D+T_{P}$$ (2)

where η_S_ is the solvent viscosity, **D *=*** 0.5((∇**v**) + (∇**v**)^T^) is the rate of deformation with **v** as the velocity vector, and **T**_P_ is the upper convicted polymeric stress, calculated from

$$\lambda_{P}(\frac{\partial }{\partial t}T_{P}+v∙\nabla T_{P}-{\left(\nabla v\right)}^{T}∙T_{P}-T_{P}∙(\nabla v))+T_{P}=2\eta_{P}D$$ (3)

with λ_P_ and η_P_ being the polymer relaxation time and viscosity, respectively. The single-mode Oldroyd-B model captures the viscoelastic effect while assuming a steady shear viscosity for the fluid. The microstreaming velocity field and the corresponding particle trajectory indicate different values and patterns for each fluid type. The generated flow vortex is dominant in the Newtonian fluid (such as water), reduces relatively for shear-thinning cases, and diminishes for viscoelastic fluids. Notably, the shear rate upon oscillation of the microbubble is considerably high in front of the microrobot’s orifice, reaching up to $\overset{\cdot }{\gamma }$ ~ 10^2^ s^−1^ and $\dot{\gamma }$ ~ 10^3^ s^−1^ in viscoelastic and pure shear-thinning fluids, respectively. The simulation results of tracer particle trajectories match well with the experimental results, where the tracer particles were used to show the streamlines in the Newtonian fluid (PBS in this case) under acoustic actuation at 380 kHz (Fig. 3B). The average velocity of the tracer particles for a range of excitation frequencies between 300 and 500 kHz was obtained using a custom-made tracking script, as shown in fig. S4. The average particle speeds peaked at around 380 kHz and dropped at other frequencies. However, because of the overdamped oscillatory response of the microbubble, the frequency bandwidth is wide such that enough thrust can be generated at other frequencies.

We tested the performance of the microrobots immersed inside mucus. An array of microrobots were actuated at 380 kHz and 1 V_pp_ driving voltage. Immediately, upon ultrasound actuation, the local microstreaming was generated (Fig. 3C and movie S2). The polymeric network of mucus was deformed by following the microstreaming flow in front of the microrobot, similar to the simulated particle trajectory results inside the viscoelastic fluid in Fig. 3A. However, the surrounding mucus medium far from the front region of the oscillatory microbubble was stationary. The local disturbance and deformation of the mucus polymeric structure together with the high shear rate thrust force of the microrobot can cause the local phase separation of mucus that leads to a low-viscosity layer nearby the microrobot (*46*).

### Propulsion of the acoustic microrobots inside mucus

**Fig. 4.**
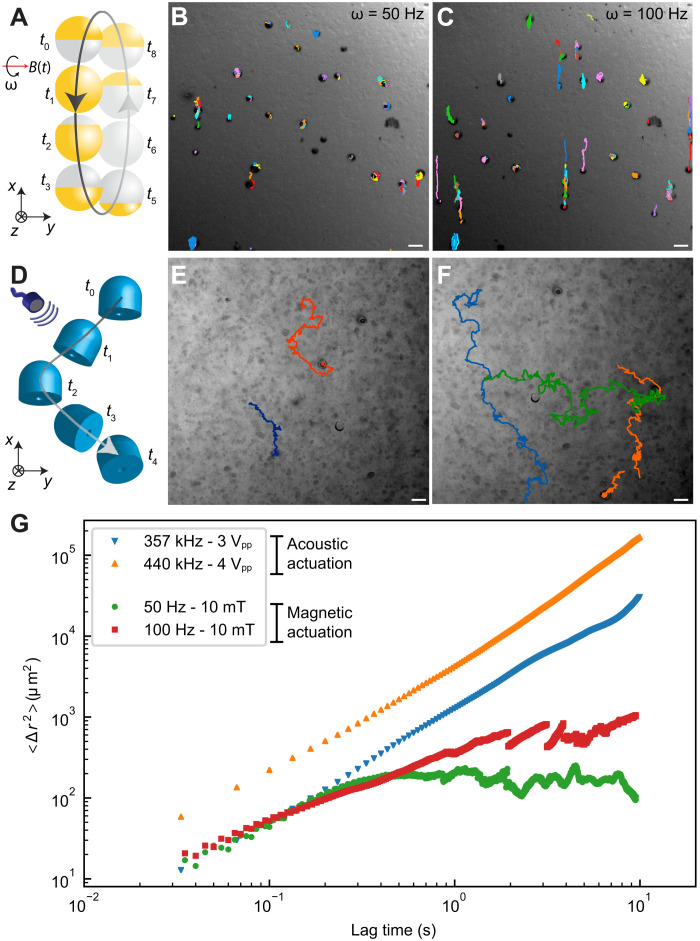
Comparison of high shear rate propulsion of the acoustic microrobots with the surface-rolling motion of the spherical magnetic microrobots inside mucus. (**A**) The schematics of the surface-rolling mechanism of the Janus magnetic microrobots inside mucus medium under a rotational uniform magnetic field. (**B**) The time-lapse trajectories of 25-μm-diameter magnetic microrobots inside mucus, actuated by a rotational frequency of 50 Hz and a uniform magnetic field of 10 mT. The magnetic microrobots were spinning without considerable propulsion, as they were pushed back by the viscoelastic mucus network. Increasing the rotational frequency of the magnetic microrobots to 100 Hz slightly improved their locomotion inside mucus (**C**). (**D**) The schematics of the puller-type propulsion for the acoustic microrobots inside mucus. (**E**) The time-lapse trajectories of the acoustic microrobots penetrating through mucus under 3 V_pp_ driving voltage. The random trajectories are caused by the heterogeneous mucus microstructure. (**F**) Enhanced locomotion trajectories of the acoustic microrobots under 4 V_pp_ driving voltage. Scale bars, 50 μm. (**G**) The experimental ensemble mean square displacement (MSD) results as a function of the lag time for the acoustic and magnetic microrobots. The MSD values for the acoustic microrobots indicate superior locomotion over the magnetic microrobots inside mucus. The single magnetic microrobots showed back-and-forth motion due to mucus resistance, which is captured by the MSD values after around 1-s lag time.

We demonstrated that the high shear rate streaming flow could generate local deformations in the polymeric network of mucus. For comparison, we tested a recently developed microrobotic system with a similar size (25 μm in diameter). Under a rotating uniform magnetic field, the spherical Janus magnetic microparticles use surface rolling for locomotion (*11*). In a selected rolling-based microrobot system, the rotational speed can be precisely controlled by an externally applied magnetic field. The surface microrollers, injected in the mucus medium, were actuated under a 10-mT magnetic field and rotational frequency of 50 to 100 Hz. Figure 4A schematically illustrates the magnetically actuated microroller trajectories inside mucus. The microparticles at 50 Hz were spinning without considerable locomotion (Fig. 4B). By increasing the rotation frequency to 100 Hz, they were rolling for a small path and then pushed back to their original position by the mucus (Fig. 4C and movie S3). This locomotion pattern could be attributed to the viscoelastic nature of the mucus, acting as a resistive force pushing back the particle. In contrast, the acoustically actuated microrobots were not pushed back inside the mucus (schematically depicted in Fig. 4D) and continued their puller-type propulsion in a random trajectory, shown in Fig. 4 (E and F) and movie S3. During the locomotion, the orientation of the microrobots with the orifice remained parallel to the substrate (Fig. 4D).

We observed that the microrobots self-aligned at some regions perpendicular to the substrate and then switched back to the horizontal orientation during the actuation. This behavior could be attributed to the interplay between the second-order Bjerknes force, which attracts the oscillating microbubble toward the surface, and the thrust force inside the heterogenous mucus structure. That is, in some regions, the nonlinear viscoelastic stresses generated by the microstreaming flows causes the symmetry breaking and hence propulsion of the microrobots; in addition, in diluted liquid-like areas of the mucus network, the Bjerkness force dominates and rotates the microrobot axis perpendicular to the substrate. Regarding the direction control of the microrobots in mucus, a magnetic film coating on the microrobot surface could enable its magnetic steering, as shown previously (*16*). By using only ultrasound actuation, two microbubbles with different resonant frequencies could be also possible, as recently demonstrated for Newtonian fluids (*47*). However, the presence of non-Newtonian fluids and consequently the change of propulsion mode would require a complex multibubble design.

Last, to compare the propulsion performance of the two microrobotic systems, we calculated the ensemble mean square displacement (MSD) of the microrobots as shown in Fig. 4G. The ensemble MSD, as a metric for comparing the activity of the microrobots during actuation, shows that the acoustic microrobots could successfully move inside the mucus at larger lag times. The magnetic microrollers, however, failed to continuously move through mucus, which is verified by the plateau MSD region at higher lag times.

### In vitro demonstration of the acoustic microrobot propulsion on cellular topographical surfaces

**Fig. 5.**
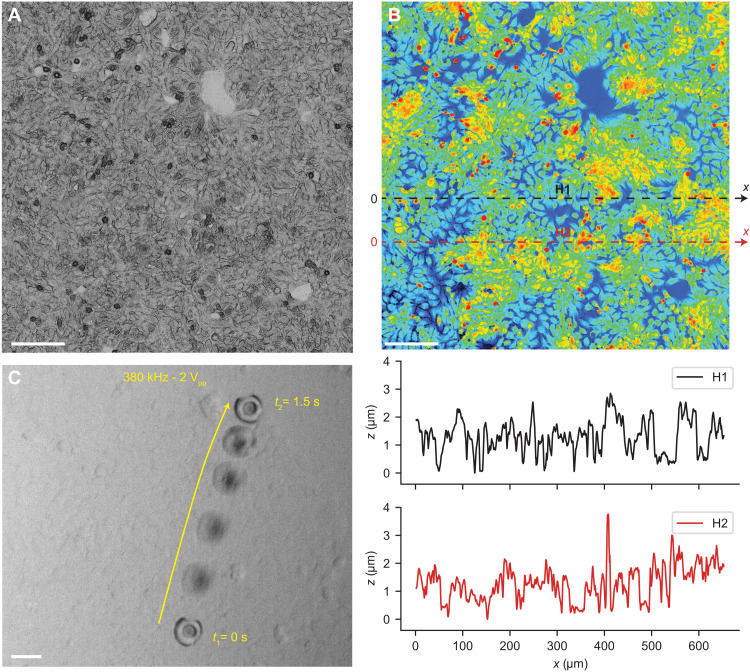
Propulsion of the acoustic microrobots on the 3D topographical surface of the epithelium cellular layer in vitro. (**A**) Confocal microscopy image of the epithelium layer that was used as a 3D topographical surface for locomotion of microrobots. Scale bar, 100 μm. (**B**) Height heatmap of the epithelium layer surface measured by a confocal microscope, with a peak height of the cell nuclei between 2 and 4 μm. Scale bar, 100 μm. The height profile for two representative lines, denoted by H1 and H2, depicts the complex 3D topography of the cell layer. (**C**) Time-lapse images of the acoustic microrobot propulsion on the epithelium cells during 1.5 s, under ultrasound actuation of 380 kHz and 2 V_pp_. Scale bar, 30 μm.

In addition to the rheological behavior of the complex biofluids, the surface topography of the cells becomes important for the locomotion of microrobots (*48*). For example, the mucus is secreted by the epithelium, which has a certain 3D topography in different organs. To test the acoustic microrobots under physiologically realistic conditions, we developed an epithelial cell layer found in the colon in vitro (Fig. 5A). We measured the topographical profile of the cells with nucleus heights ranging between 2 and 4 μm (Fig. 5B). The mucus medium with the acoustic microrobots was injected onto the cell layers. Immediately after ultrasound activation, the microrobot started shaking on the cell layers. This behavior before locomotion is due to the partial nonspecific attachment to the cell surface, while it creates the microstreaming flow in front of the nozzle of the microrobots. Once the propulsion thrust overcame the adhesion to the cell layer, the microrobots were detached and propelled on the cell layer with a tilted orientation (Fig. 5C and movie S4). To decrease the adhesion of the microrobots to the biological cells, certain surface treatments, such as perfluorocarbon coating, could be used (*28*). Note that, as long as the microrobots are moving on tissues/cells, the microstreaming thrust is consumed for propulsion and thus would not have any side effects. However, if the microrobots are fixated such that the microbubble interface is very close to the cells/tissues, then the high shear rate could exert mechanical strain on the cells. This interesting behavior can be used to have microsurgery applications in the case of cancer cell lysis or sonoporation (*49*).

## DISCUSSION

We presented a high shear rate propulsion mechanism using acoustic microrobots inside biological fluids. For Newtonian fluids with a steady shear viscosity, the secondary order Bjerknes force was dominant, leading to a surface-slipping motion. However, for viscoelastic shear-thinning biofluids, such as blood and mucus, the interplay between the Bjerknes force and the thrust force led to puller-type propulsion. Through numerical simulations, we showed that the robot’s bubble oscillation–based microstreaming pattern is highly correlated to the rheological property of the fluid. For the extreme case of mucus, we observed that the generated microstreaming flow exerted enough momentum to the medium such that the polymeric network was locally deformed in front of the microrobot. Once freed, the microrobots under ultrasound actuation could locally disrupt the mucus and propel in a puller-type fashion. Moreover, we demonstrated the significance of high shear strain by comparing it to the surface-rolling microrobot system inside the mucus medium. At lower rotation frequencies, i.e., lower shear rate strains, the magnetic microrobots were unable to move through the mucus network and were repeatedly pushed back to their original position. Although increasing the rotational frequency helped in increased translation of the single magnetic microrobots, their continuous propulsion still became futile. In contrast, the acoustic microrobots could continuously move through the mucus polymeric network, thanks to their high shear rate streaming flow generation. Last, to mimic a biological scenario where mucus is secreted by epithelium cells, we demonstrated the locomotion of the acoustic microrobots on topographical cell layers in presence of the mucus medium.

A direct implication of this study could be in enhancing microrobotic drug delivery efficiency inside viscoelastic biofluids, such as mucus, which is ubiquitous inside our body. Current active microrobotic and passive drug delivery systems are known to have low therapeutic efficiency in penetrating the mucus layer and reaching the epithelium cells (*23*). Integrating cargos, such as drugs, into these acoustic microrobots could be a solution to enhance the mucus-penetrating character of the drug delivery systems in a controlled and local manner. For example, the drug-loaded acoustic microrobots could be locally actuated at the desired area when the ultrasound probe of a certain diameter is aligned and coupled externally to the tissue, and incident waves are transmitted to the target site.

Another application of the proposed acoustic microrobotic system would be in the characterization of the viscoelastic biofluids as an active local microrheology tool (*50*, *51*). Recent micro- and nanorobotic studies have shown the great potential of small-scale mechanical probes for sensing the local rheological property of heterogeneous environments within low to moderate shear rates (*52*–*60*). In this vein, the rheological properties of heterogeneous biofluids, especially at higher strain rates (>10^3^), could be characterized using the proposed acoustic system, which is a hurdle in bulk rheological measurements. Complimentary to the microrheology techniques using free bubbles (*49*, *50*), one could use the cavity designs in our study to trap bubbles of different size scales (~1 to 1000 μm) and perform statistical tests to fit constitutive laws for various biofluids. The controlled bubble size enforced by the cavity design would be advantageous over free bubbles, as their resonance frequency and forced oscillation behavior can be precisely controlled.

## MATERIALS AND METHODS

### Fabrication of the acoustic microrobots

The acoustic microrobots were 3D printed using a commercially available two-photon polymerization system (Photonic Professional GT, Nanoscribe GmbH, Karlsruhe, Germany) with a 63× oil immersion objective (numerical aperture = 1.4). The double reentrant cavity designs were printed on indium tin oxide (ITO)–coated glass substrates using IP-Dip photoresin (Nanoscribe GmbH, Karlsruhe, Germany), enabling the submicrometer printing resolution and high aspect ratio features. After the 3D printing step, the microrobot arrays were developed in propylene glycol monomethyl ether acetate (Sigma-Aldrich Inc., St. Louis, MO, USA) for 45 min, followed by a rinse in isopropyl alcohol. The 3D profile of double reentrant microstructures, vital for liquid repellency, were characterized in half-printed microrobots using a 3D laser scanning microscopy (VK-X200, KEYENCE).

The Janus spherical magnetic microrollers were fabricated by the procedure detailed in the previous work (*11*). Briefly, a monolayer of silica (SiO_2_) particles of 25 μm in diameter (Microspheres-Nanospheres, Cold Spring, NY, USA) was prepared under a dry condition. The magnetic nanofilm was deposited by sequentially sputtering Ni (1000 nm in thickness) and Au (20 nm in thickness), using a benchtop sputter coating system (Leica EM ACE600, Leica Microsystems). After creating the Janus microparticles, their magnetization direction was set to out-of-plane direction by applying a 1.8-T uniform magnetic field in a vibrating sample magnetometer (MicroSense, Lowell, MA). Last, the Janus microparticles were released from the glass substrate by sonication in ethanol solution.

### Acoustic actuation and pressure measurement systems

A planar immersion ultrasound probe with the center frequency of 500 kHz (A301S-SU, Olympus Deutschland GmbH) was used for actuation (fig. S6). The probe was immersed in a water tank and directed with a 45° angle toward a PDMS chamber containing the microrobots. The PDMS chamber consisted of a circular wall with a sealed cap to isolate the biological medium and the microrobots from the surrounding DI water. A sinusoidal driving signal was generated using an arbitrary function generator (AFG3102C, Tektronix Inc.), amplified 50 times by a piezo amplifier (Model 2100HF, Trek Inc.), and sent to the ultrasound probe for actuation. The typical driving voltage amplitude and frequency were in the range of 1 to 4 V_pp_ and 350 to 480 kHz, respectively. For acoustic pressure measurements, a calibrated needle hydrophone of 500-μm tip diameter (NH0500, Precision Acoustics Ltd.) was placed in the middle of the chamber and above the glass substrate. The time domain signals from the driving voltage and acoustic pressure were recorded using a mixed domain oscilloscope (MDO4024C, Tektronix Inc.). Then, the collected signals were analyzed using a Python script, and the peak amplitudes for the driving voltage and the corresponding acoustic pressure amplitude at different frequencies were obtained.

### Magnetic actuation setup

The magnetic microrobots were actuated using a custom-made electromagnetic coil setup. The coil system was mounted on an inverted optical microscope (Zeiss Axio observer A1, Carl Zeiss, Oberkochen, Germany) for simultaneous actuation and imaging. Individual coils were controlled independently by a current controller setup (Escon 70/10, Maxon Motor AG). For actuation of 25 μm of Janus particles, a 10-mT rotating magnetic field with frequencies up to 100 Hz was used.

### Imaging and tracking of the microrobots

The acoustic microrobots were imaged in an inverted optical microscope (Eclipse Ti-E, Nikon Instruments Inc.). The propulsion of the acoustic microrobots inside biofluids was captured by Hamamatsu Orca Flash4 camera (Hamamatsu Photonics) at 30 frames/s (fps). For characterization of the microbubble resonance, 2-μm tracer particles were injected in the medium to follow the microstreaming trajectories, and images were taken by a high-speed camera system (M310, Phantom Inc.) at 1000 fps. The images of the magnetic rollers rotating at 50 and 100 Hz inside mucus were also recorded by the high-speed camera at 200 fps. The tracking of microrobots was all performed using a custom-made Python script using either the Trackpy package in Python (*61*) or Manual Tracking in Fiji (*62*) for feature detection and linking trajectories. For finding the average instantaneous speed of acoustically actuated microrobots, we used a velocity averaging technique over neighboring time steps (*63*). That is, for a given center of mass position of each microrobot {*x_j_*} with *x_j_* = (*x*(*t_j_*), *y*(*t_j_*)) for *j* = 1, …, *M* time steps, the speed at a given time *t_j_* was calculated as

$$V_{j,n}=\frac{1}{n∆t}\|x_{j+\frac{n}{2}}-x_{j-\frac{n}{2}}\|$$ (4)

where ∆*t* is the time interval between two consecutive frames and *n* is the neighboring time steps chosen as 10 steps in our study, to avoid sudden speed fluctuations. To remove the stopping events (due to adhesion to the substrate) from the average speed calculation, arithmetic mean from values of {*V*_*j*,*n*_}, which satisfied *V*_*j*,*n*_ > 0.5 max {*V*_*j*,*n*_}, was taken. The ensemble MSD for *N* number of particles was calculated from

$$<r^{2}>=\frac{1}{N}\sum_{i=1}^{N}{|x^{\left(i\right)}(t)-x^{i}(0)|}^{2}$$ (5)

### Acoustic microstreaming simulations

COMSOL Multiphysics 5.6 (COMSOL Inc.) was used to calculate the acoustic microstreaming field around the microrobot. The microbubble interface with a fluid domain was defined as an oscillatory boundary layer with a sinusoidal function. To predict the microstreaming flow characteristics during the oscillation of the microbubble, a 2D axisymmetric simulation was conducted. The acoustic fields in the fluid domain were calculated by solving the first- and second-order perturbation equations (*8*, *64*). The governing thermoacoustic equations for the first-order temperature (*T*_1_) and pressure field (*p*_1_) and the dynamic Navier-Stokes equation for first-order velocity field (**v**_**1**_) are (*64*, *65*)

$$\begin{array}{c} i\omega T_{1}=i\omega \frac{\alpha T_{f}}{\rho_{f}C_{p}}p_{1}-D_{\text{th}}\nabla^{2}T_{1}\\ i\omega p_{1}=\frac{1}{\gamma \kappa_{f}}[i\omega \alpha T_{1}+\nabla \cdot v_{1}]\\ i\omega \rho_{f}v_{1}=\nabla p_{1}-\eta \nabla^{2}v_{1}-\beta \eta \nabla (\nabla \cdot v_{1}) \end{array}$$ (6)

where *D*_th_, γ, α, η, *C*_p_, and ω are the thermal diffusivity, specific heat capacity ratio, thermal expansion coefficient, viscosity, specific heat capacity, and angular frequency, respectively. The temperature of the fluid (*T*_f_) is 25°C before the presence of an acoustic wave. The density of the fluid is ρ_f_, and β is the viscosity ratio. The second-order time-averaged continuity equation and Navier-Stokes equation are given by (*64*, *65*)

$$\begin{array}{c} \rho_{f}\nabla \cdot \langle v_{2}\rangle =-\nabla \cdot \langle \rho_{1}v_{1}\rangle \\ \eta \nabla^{2}\langle v_{2}\rangle +\beta \eta \nabla (\nabla \cdot \langle v_{2}\rangle )-\langle \nabla p_{2}\rangle =\langle \rho_{1}\delta_{t}v_{1}\rangle +\rho_{f}((v_{1}\cdot \nabla )v_{1}\rangle \end{array}$$ (7)

where ⟨**v**_**2**_⟩ is the streaming velocity as shown in Fig. 3A. Note that, for the shear-thinning fluids, the viscosity is not constant and is defined by the Carreau model in Eq. 1. Moreover, for the viscoelastic fluids, which is described by the single-mode Oldroyd-B model in our study (Eqs. 2 and 3), an extra stress tensor (**T**_E_) is added to the total stress (**σ**) in the Navier-Stokes equation as

$$\sigma =-p_{2}I+\eta_{s}\nabla v_{2}+\beta \eta_{s}(\nabla \cdot v_{2})+T_{E}$$ (8)

The boundary conditions for the computational domain were set by **v** = ±*v*_0_ **n**, where *v*_0_ is the velocity amplitude of microbubble oscillation defined as *v*_0_ = *A*ω, assuming a harmonic motion of the bubble boundary (*Ae*^*j*ω*t*^), and **n** indicates the normal-to-surface of the bubble direction. Here, we assumed the oscillation amplitude of the microbubble as *A* = 500 nm and 380 kHz as the excitation frequency. For the solid-fluid interface, no-slip boundary conditions and **v** = 0 at the solid boundary, assuming no deformation of microrobot’s rigid shell, were defined.

The above equations were numerically solved within the thermoviscous acoustics and laminar flow interfaces of the COMSOL Multiphysics software. The thermoviscous acoustics interface was used to solve the first-order perturbation equations in Eq. 6, and sequentially, the laminar flow interface was used to solve the second-order perturbation equations in Eq. 7. The computed microstreaming velocity field and the shear rate strain field were shown in Fig. 3A for Newtonian, shear-thinning, and viscoelastic fluids.

To predict the particle trajectory during the oscillation of microbubble, the particle tracing model in COMSOL Multiphysics was used. For this, the forces acting on single particles (2-μm-diameter tracers) including the acoustic radiation force (**F**^rad^), the drag force (**F**^drag^), and the gravitational force (**F**^grav^) should be considered. The acoustic radiation equation was calculated by (*65*) as

$$F^{\text{rad}}=-\pi a^{3}[\frac{2}{3}\text{Re}[f_{1}^{*}p_{1}^{*}\nabla p_{1}]-\rho_{f}\text{Re}[f_{2}^{*}v_{1}^{*}∙\nabla v_{1}]]$$ (9)

where $\kappa_{f}=1/(\rho_{0}c_{0}^{2})$is the compressibility of the fluid and *Re* and the asterisk (^*^) denote the real and the complex conjugate part of the parameter, respectively. The monopole coefficient *f*_1_ and the dipole coefficient *f*_2_ were given by

$$f_{1}=1-\frac{\kappa_{p}}{\kappa_{f}}\;\text{and}\;f_{2}=\frac{2(\rho_{p}-\rho_{f})}{2\rho_{p}-\rho_{f}}$$ (10)

where κ_p_ and ρ_p_ are the compressibility and the density of the particle, respectively. The time-averaged Stokes drag force on a spherical particle was given by (*64*)

$$F^{\text{drag}}=6\pi \eta a(\langle v_{2}\rangle -v_{p})$$ (11)

where *a* and **v**_p_ are radius and velocity of the particles, respectively. The gravitational force by accounting for the buoyancy of particles was calculated as $F^{\text{grav}}=\frac{4}{3}\pi a^{3}(\rho_{f}-\rho_{p})g$. Considering Newton’s second law of motion and neglecting the inertia of particles, the time-averaged velocity of particles was calculated as

$$v_{p}=\langle v_{2}\rangle +F^{\text{rad}}+\frac{F^{\text{grav}}}{6\pi \eta a}$$ (12)

### Preparation and rheology of the complex biofluids

Newtonian fluids with different viscosities were prepared by mixing DI water with glycerol (Sigma-Aldrich) at different concentrations. We studied microrobot propulsion in Newtonian Gl-DI mixtures at 20, 40, and 60% (w/w) concentrations with viscosities of 1.6, 3.5, and 10.1 cP, respectively. FBS was used as a non-Newtonian shear-thinning biofluid model. Whole blood from mice was used as a non-Newtonian shear-thinning granular biofluid model. Both FBS and mouse blood were provided by Einrichtung für Tierschutz, Tierärztlichen Dienst und Labortierkunde, and Eberhard Karls University Tübingen.

Synthetic mucus was used as a viscoelastic biofluid model. Mucus was prepared by adding 6% (w/v) of pig gastric mucin type III (Sigma-Aldrich) and 1% (w/v) of bovine serum albumin (Sigma-Aldrich) to an aqueous buffer at pH 7.4 (154 mM NaCl, 3 mM CaCl_2_, and 15 mM NaH_2_PO_4_/Na_2_HPO_4_) (*66*). The mucin solution was homogenized and mixed thoroughly for 24 hours. To cross-link the mucin solution, 10% (w/w of mucin) of glutaraldehyde (Sigma-Aldrich) was added to the mixture and was left cross-linking for 24 hours.

The viscosity and viscoelasticity of the fluids were characterized in a TA Instruments DHR 30 rheometer using a 40-mm, 1° cone plate. Steady shear experiments were performed to measure the viscosity as a function of shear rate from 1000 to 0.01 s^−1^ with an averaging time of 30 s per point. Oscillatory experiments were performed to measure the shear moduli as a function of frequency from 0.1 to 100 rad/s at 2% strain.

### Epithelium cell culture

HT-29, epithelial cell line was obtained from the American Type Culture Collection (Rockville, MD, USA). The cells were cultured in Dulbecco’s modified Eagle’s medium supplemented with 10% (v/v) FBS, penicillin (50 UI/ml), and streptomycin (50 μg/ml) in a humidified, 37°C, 5% CO_2_ environment using 75-cm^2^ polystyrene cell culture flasks. For the mucus experiments, the cells were subcultured to fibronectin-coated glass slides and cultured for at least 3 days to reach confluence. After reaching confluence, cells were fixed using 4% paraformaldehyde to keep the topography of the cells stable during the locomotion experiments.

### Statistical analysis

The quantitative values were presented as one SD of the mean.
