# Supplementary material for: High shear rate propulsion of acoustic microrobots in complex biological fluids
Source: Sci Adv. 2022 Mar 11;8(10):eabm5126. doi: 10.1126/sciadv.abm5126 (PMC8916727; doi:10.1126/sciadv.abm5126)
Supplement: Supplementary file 1 — Figs. S1 to S8 [file sciadv.abm5126_sm.pdf]

Science Advances
AAAS

Supplementary Materials for

High shear rate propulsion of acoustic microrobots in complex biological fluids

Amirreza Aghakhani, Abdon Pena-Francesch, Ugur Bozuyuk, Hakan Cetin, Paul Wrede, Metin Sitti*

*Corresponding author. Email: sitti@is.mpg.de

Published 11 March 2022, *Sci. Adv.* **8**, eabm5126 (2022)
DOI: 10.1126/sciadv.abm5126

The PDF file includes:

Figs. S1 to S8

Other Supplementary Material for this manuscript includes the following:

Movies S1 to S4

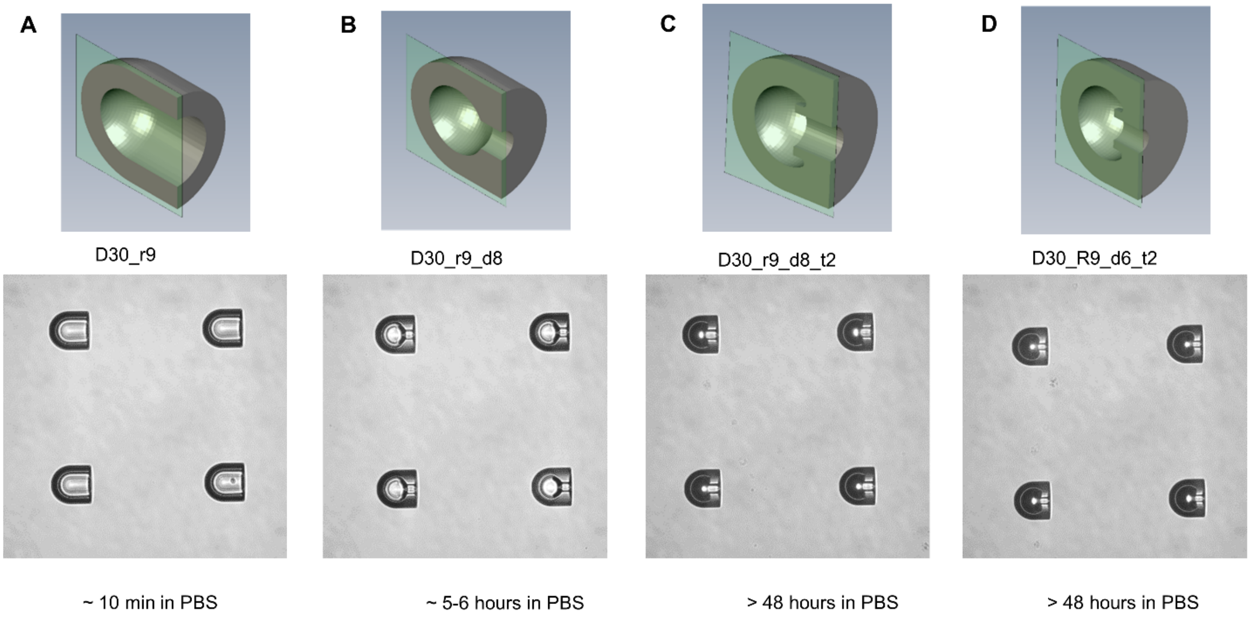

Fig. S1. Bubble stability tests in different coating-free cavity designs of different acoustic microrobot designs. (A) The cylindrical cavity design has the worst bubble holding capability because the air bubble diffuses quickly in about 10 minutes as the fluidic medium (PBS in this case) wets the inner surface of the cavity. **(B)** The spherical cavity design with an orifice diameter of d = 8 μm held the bubble stable for about 5-6 hours. **(C)** – **(D)** The double re-entrant designs introduced in the spherical cavity drastically improved the bubble stability to more than 48 hours inside PBS. The volume of the trapped bubble became smaller for the orifice of d = 8 μm in (**C**) compared to d = 6 um case in **(D)**, which is why the latter design was selected as the main design throughout the paper.

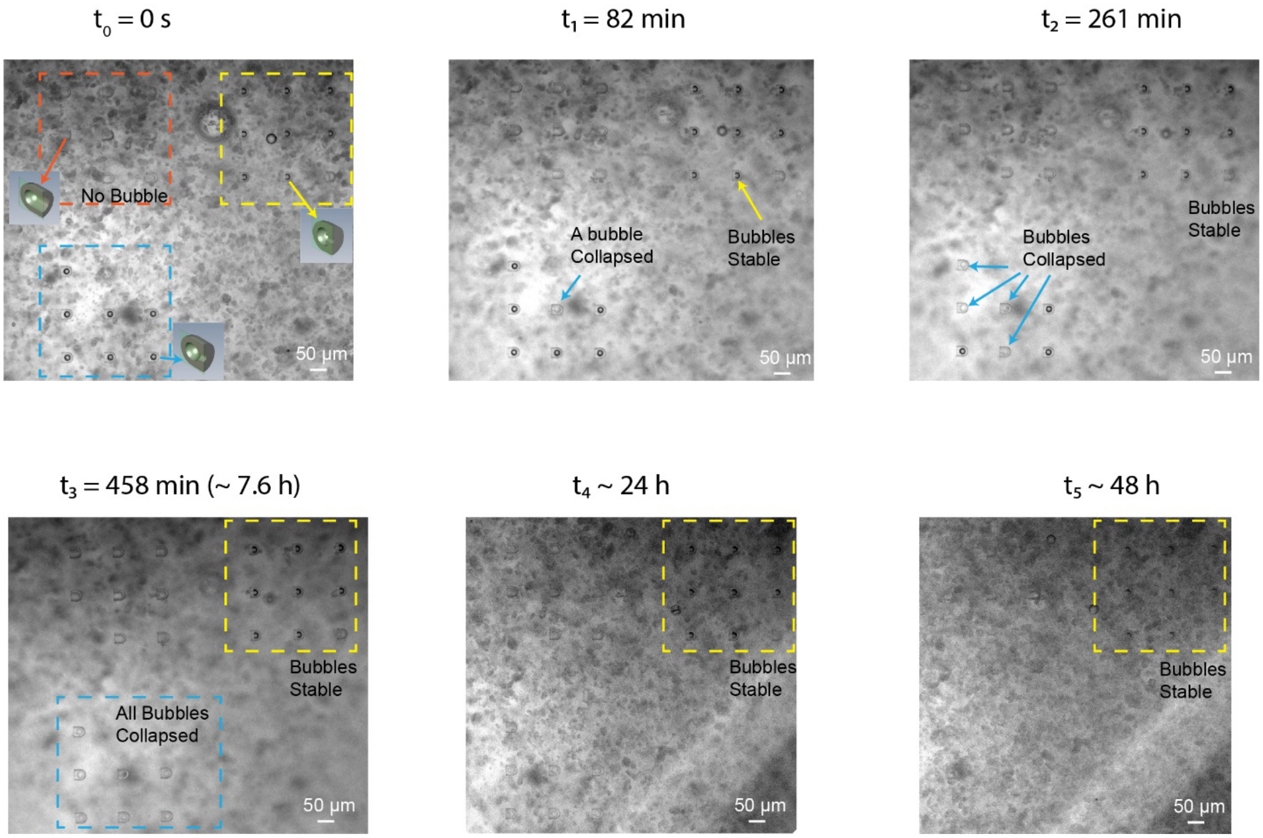

Fig. S2. Time-lapse images of the bubble stability test for different microrobots in mucus. Immediately after injection of mucus biofluid, no bubble was trapped inside the cylindrical cavity (indicated by the orange arrow), whereas, bubbles were trapped firmly inside the standard spherical cavity (blue arrow) and double reentrant spherical cavity (yellow arrow). The standard spherical cavity designs started losing their bubble in time, and finally, within about 7.6 hours, all the bubbles were collapsed. The double reentrant designs could hold the bubbles for about 48 hours, about six times better performance than the standard spherical cavity design.

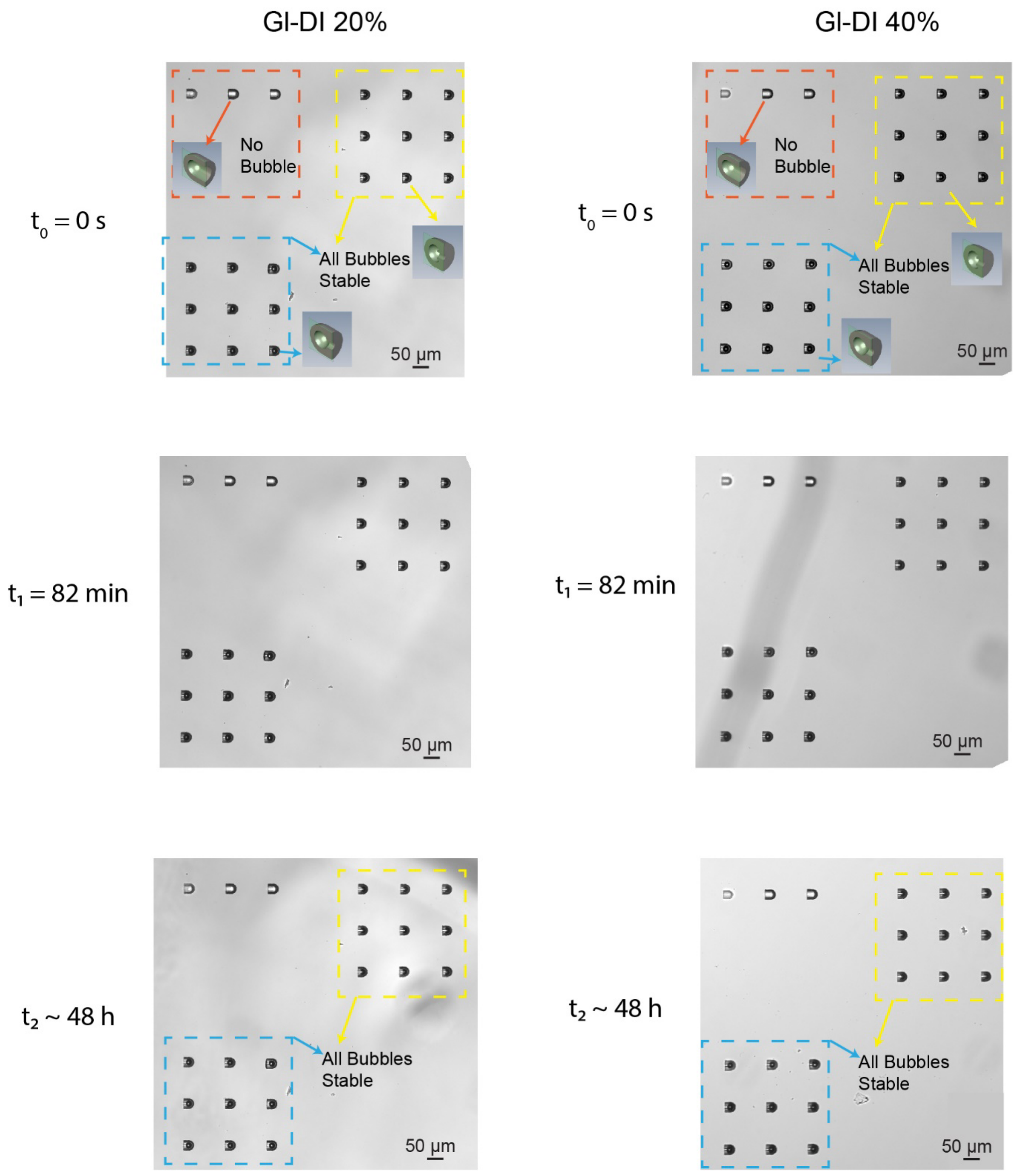

Fig. S3. Time-lapse images of the bubble stability test for different microrobots in glycerol-DI water ratios of 20% and 40%. Immediately after injection of GL-DI fluid, no bubble was trapped inside the cylindrical cavity (indicated by the orange arrow), whereas, bubbles were trapped firmly inside the standard spherical cavity (blue dashed line) and double reentrant spherical cavity (yellow dashed line). Interestingly, both spherical and double reentrant cavity designs could hold microbubbles for about 48 hours, as a result of low gas diffusion in the GL-DI mixture.

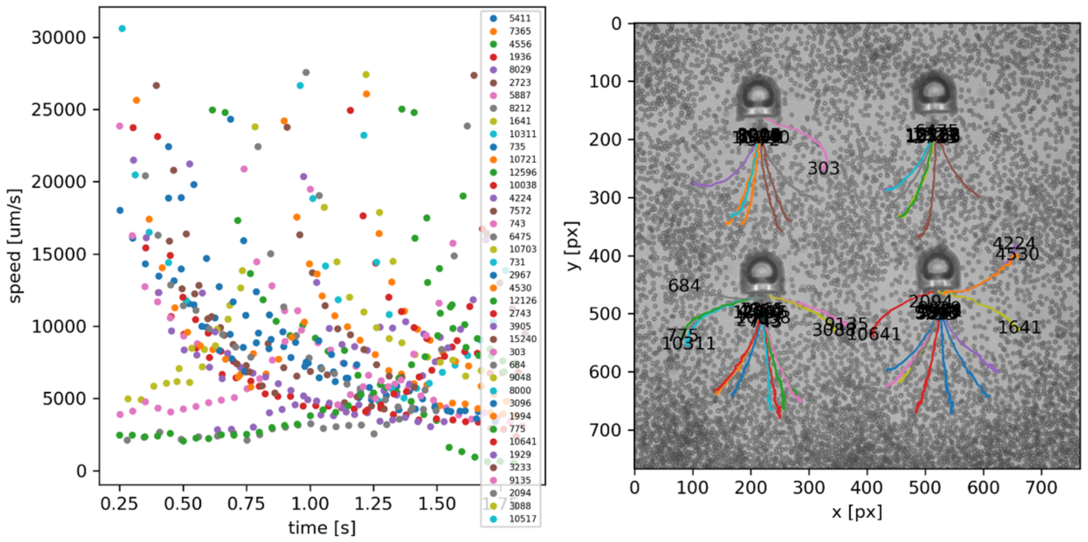

Fig. S4. Speed results of the individual tracer particles in front of the acoustically actuated microrobots. The diagram on the left shows the speed of individual tracer particles, tracked frame by frame for an array of four microrobots (shown in the right panel) actuated at 380 kHz and 1 V_{pp}.

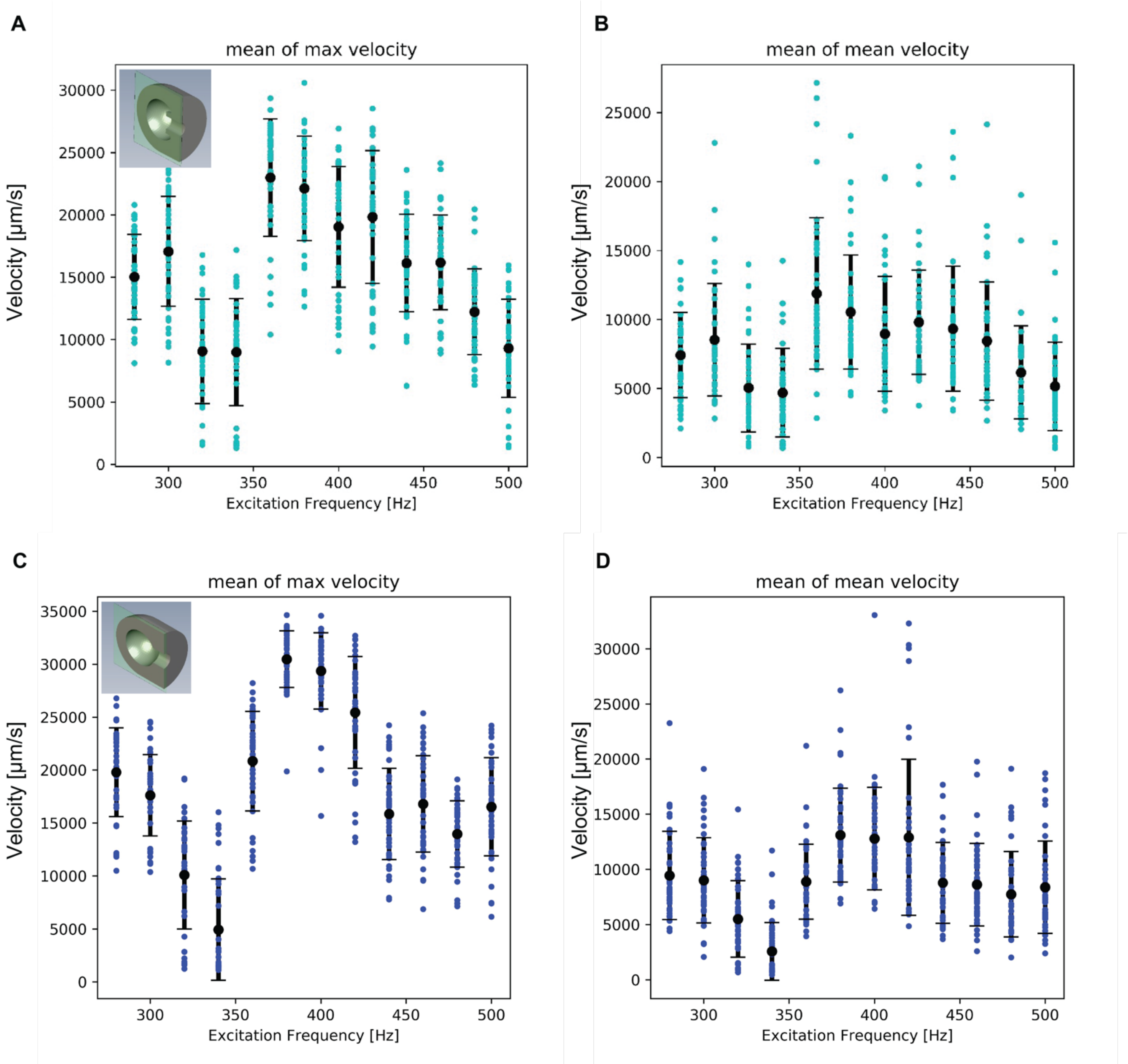

Fig. S5. Characterization of the resonance frequency of the microrobots' microbubble. (A) The mean of maximum particle velocity in front of the actuated microrobot for the *double-reentrant* cavity design at 380 kHz. **(B)** The mean of mean velocity for particles for the *double-reentrant* cavity design. **(C)** The mean of maximum particle velocity in front of the actuated microrobot for the *spherical* cavity design at 380 kHz. **(D)** The mean of mean velocity for particles for the *spherical* cavity design. The error bar represents one standard deviation. The speed of 2-µm tracer particles was calculated from an array of four microrobots fixed on the substrate as shown in Fig. S4.

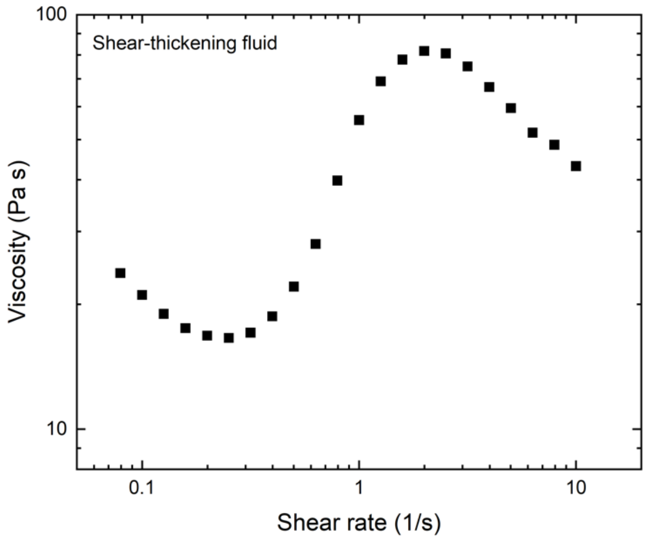

Figure S6. **Viscosity as a function of the shear rate of a shear-thickening fluid.**

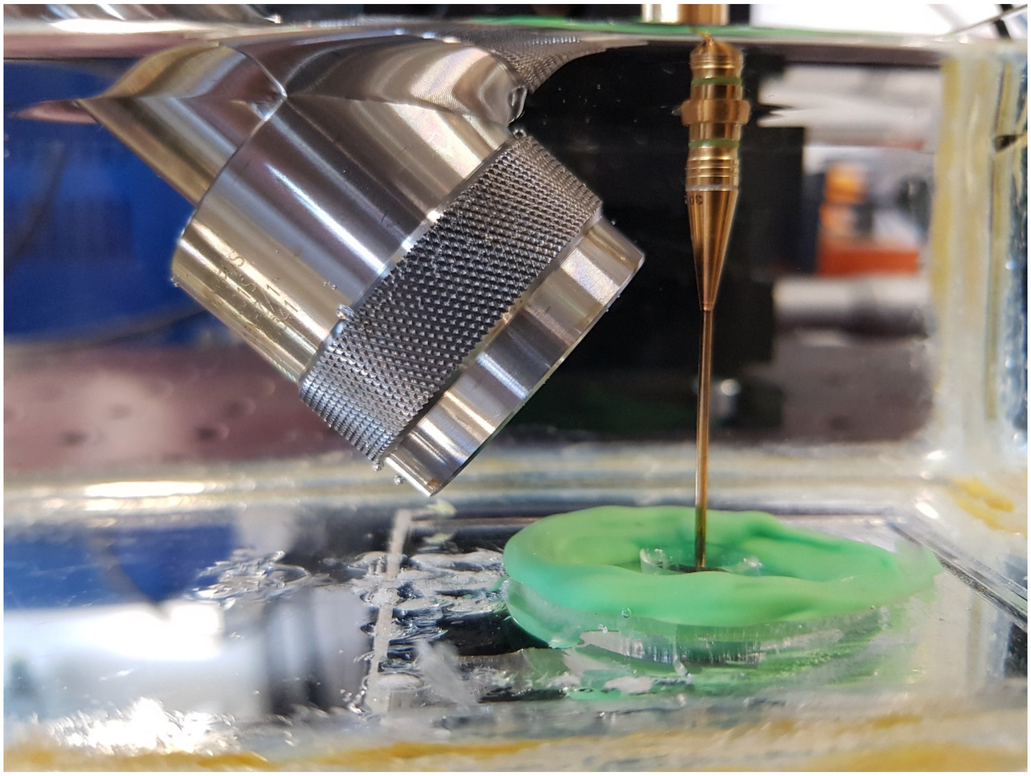

Fig. S7. Photo of the experimental acoustic setup. The ultrasound probe (left) transmits the acoustic waves of certain amplitude and frequency to the test chamber. The needle hydrophone (right) measures the acoustic pressure.

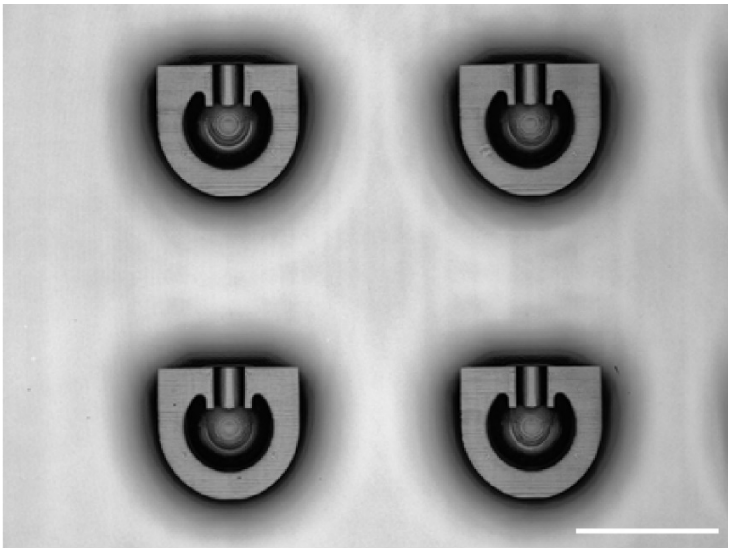

Fig. S8. The laser confocal image of the double-reentrant geometrical design of the microrobots. The scale bar is 30 μm.
